# Lévy walk dynamics explain gamma burst patterns in primate cerebral cortex

**DOI:** 10.1038/s42003-021-02256-1

**Published:** 2021-06-15

**Authors:** Yuxi Liu, Xian Long, Paul R. Martin, Samuel G. Solomon, Pulin Gong

**Affiliations:** 1 School of Physics, University of Sydney, Sydney, NSW Australia; 2 ARC Centre of Excellence for Integrative Brain Function, University of Sydney, Sydney, NSW Australia; 3 Discipline of Physiology, University of Sydney, Sydney, NSW Australia; 4 Save Sight Institute, University of Sydney, Sydney, NSW Australia; 5 Department of Experimental Psychology, University College London, London, UK

**Keywords:** Neuroscience, Computational biology and bioinformatics

## Abstract

Lévy walks describe patterns of intermittent motion with variable step sizes. In complex biological systems, Lévy walks (non-Brownian, superdiffusive random walks) are associated with behaviors such as search patterns of animals foraging for food. Here we show that Lévy walks also describe patterns of oscillatory activity in primate cerebral cortex. We used a combination of empirical observation and modeling to investigate high-frequency (gamma band) local field potential activity in visual motion-processing cortical area MT of marmoset monkeys. We found that gamma activity is organized as localized burst patterns that propagate across the cortical surface with Lévy walk dynamics. Lévy walks are fundamentally different from either global synchronization, or regular propagating waves, because they include large steps that enable activity patterns to move rapidly over cortical modules. The presence of Lévy walk dynamics therefore represents a previously undiscovered mode of brain activity, and implies a novel way for the cortex to compute. We apply a biophysically realistic circuit model to explain that the Lévy walk dynamics arise from critical-state transitions between asynchronous and localized propagating wave states, and that these dynamics yield optimal spatial sampling of the cortical sheet. We hypothesise that Lévy walk dynamics could help the cortex to efficiently process variable inputs, and to find links in patterns of activity among sparsely spiking populations of neurons.

## Abstract (web summary)

Liu et al used a combination of empirical observation and modeling to investigate high-frequency (gamma band) local field potential activity in visual motion-processing cortical area MT of marmosets. They found that gamma bursts are organised as localised patterns that propagate across the cortical surface with Lévy walk dynamics and hypothesized that such dynamics could help the cortex to efficiently process variable inputs.

## Introduction

**Fig. 1:**
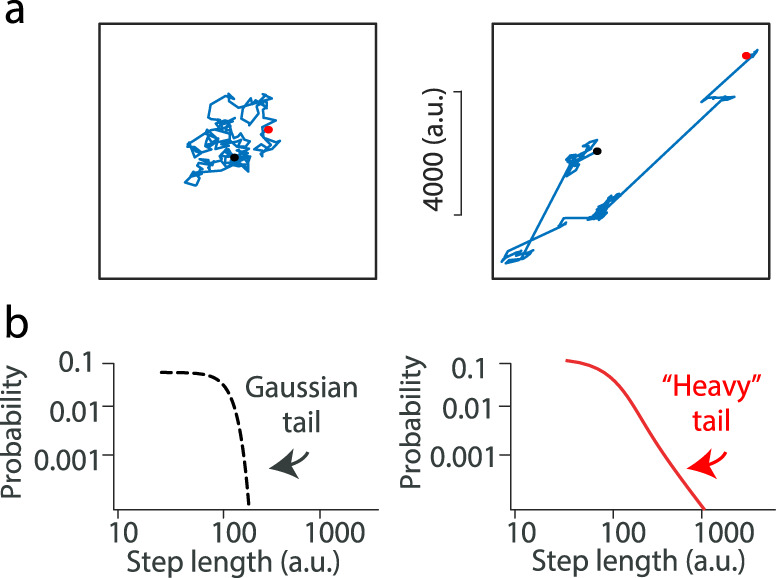
Comparison of Gaussian (Brownian) and Lévy walk dynamics. **a** Trajectory of Brownian motion (left) and that of Lévy walk (right). The initial and final positions are respectively indicated by filled red and open black circles. **b** Schematic log-log plots of the probability distributions of step lengths for Brownian motion (Left) and Lévy walk (right).

Lévy walks are anomalous diffusive forms of random motion, which have been widely observed in natural systems including movements of humans and other animals^1–6^. In a Lévy walk, clusters of short-step sizes are occasionally interspersed with longer movements. A Lévy walk therefore represents a superdiffusive process, and can travel much further from its starting position than a Brownian walk of the same duration (Fig. 1). This characteristic enables Lévy walks to efficiently implement complex tasks such as searching for sparsely distributed resources^4,7^. Recent evidence showed that Lévy walk dynamics can be intrinsically generated by neural circuits in *Drosophila* larvae^8^, but the question whether Lévy walks are a characteristic of vertebrate brain activity remains open.

Lévy walks dwell near one location for a while and then intermittently switch to new locations, leading to a “heavy-tail” probability distribution of movement step lengths (Fig. 1b). This heavy-tail property naturally generates a bursting characteristic in time and space (Fig. 1a). In the vertebrate cerebral cortex, temporal bursts of high-frequency (gamma-band) brain oscillations (30–90 Hz) have been reported, under both awake and anaesthetized conditions^9–14^. Thus the temporal properties of gamma bursts raise the possibility that they may be characterized by Lévy walks, but the spatiotemporal organization of gamma bursts remains unexplored.

Here, we first present an empirical analysis of gamma bursts in a visual motion-processing area (area MT) of marmoset cerebral cortex, under anesthesia and in absence of patterned visual stimulus (resting state). Under these conditions we find that gamma bursts typically appear as localized patterns, which propagate across the cortical surface. The propagation dynamics can be explained by Lévy walks. The occasional large displacements characteristic of Lévy walks yield wave-like propagations, and the intervening short-step clusters give rise to transient local patches of gamma synchrony. The Lévy walk dynamics of gamma burst patterns thus represent a novel mode of neural population activity.

To explain our experimental findings, we employ a spatially-extended cortical circuit model of spiking neurons^15^ to show that the Lévy walk dynamic of gamma bursts is produced by transitions between asynchronous and regular wave cortical states. We show that this mechanism allows functional interactions in the cortex across many spatial scales, without large increases in spiking rate.

The high metabolic expense of spiking activity in the brain^16,17^ means that cortical spikes are a sparsely distributed and scarce resource. We propose that Lévy walk dynamics of gamma bursts enable efficient functional linking of sparsely distributed spikes across the cortical surface, and rapid state switching to process rapid changes in the locations and timing of inputs to cortical circuits.

## Results

### Spatiotemporal dynamics of gamma bursts

**Fig. 2:**
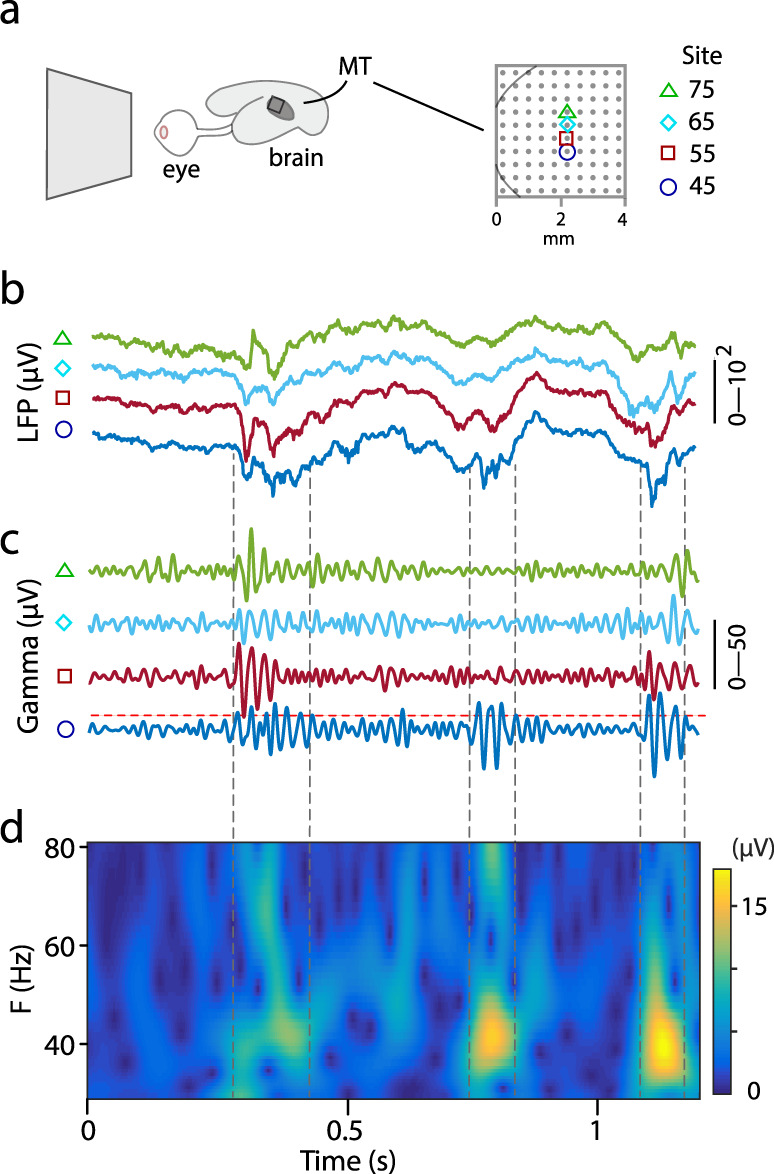
Gamma bursts recorded from marmoset cerebral cortex. **a** Schematic representation of marmoset eye and brain showing approximate position of cortical area MT and the electrode array. Magnified view of electrode array at right shows the estimated border of MT (thin gray curve) and electrode spacing. Four recording sites are indicated. **b** Broadband (0.1–500 Hz) LFP signals at the four recording sites (case MA027). **c** 30–80 Hz bandpass LFPs (gamma band) of the corresponding recording sites. Horizontal red dashed line represents power 2.5 s.d. above mean. **d** Time-frequency spectrogram of LFPs at recording site 45. Burst events are marked by gray vertical dashed lines.

We recorded local field potentials (LFPs) from the middle temporal (MT) cortical area of four sufentanil-anesthetized adult marmosets using multielectrode arrays (10 × 10 electrodes; see Methods), while the animal viewed a spatially uniform monitor screen (Fig. 2a). As expected^18^, the time-averaged power spectrum of the LFP showed bias to low temporal frequencies. Inspection of the LFP recordings at finer temporal scales, however, showed distinct bursts of power in the gamma band (30–90 Hz; Fig. 2d). These bursts were identifiable in the raw LFP traces and in the time-frequency spectrograms, computed by either bandpass filtering or wavelet decomposition (Fig. 2b–d). The burst properties are consistent with those described for spontaneous activity in mouse barrel cortex and macaque visual cortex^13,14,19^, insofar that bursts at individual electrodes exhibited variable duration and peak frequency (Fig. 2d).

**Fig. 3:**
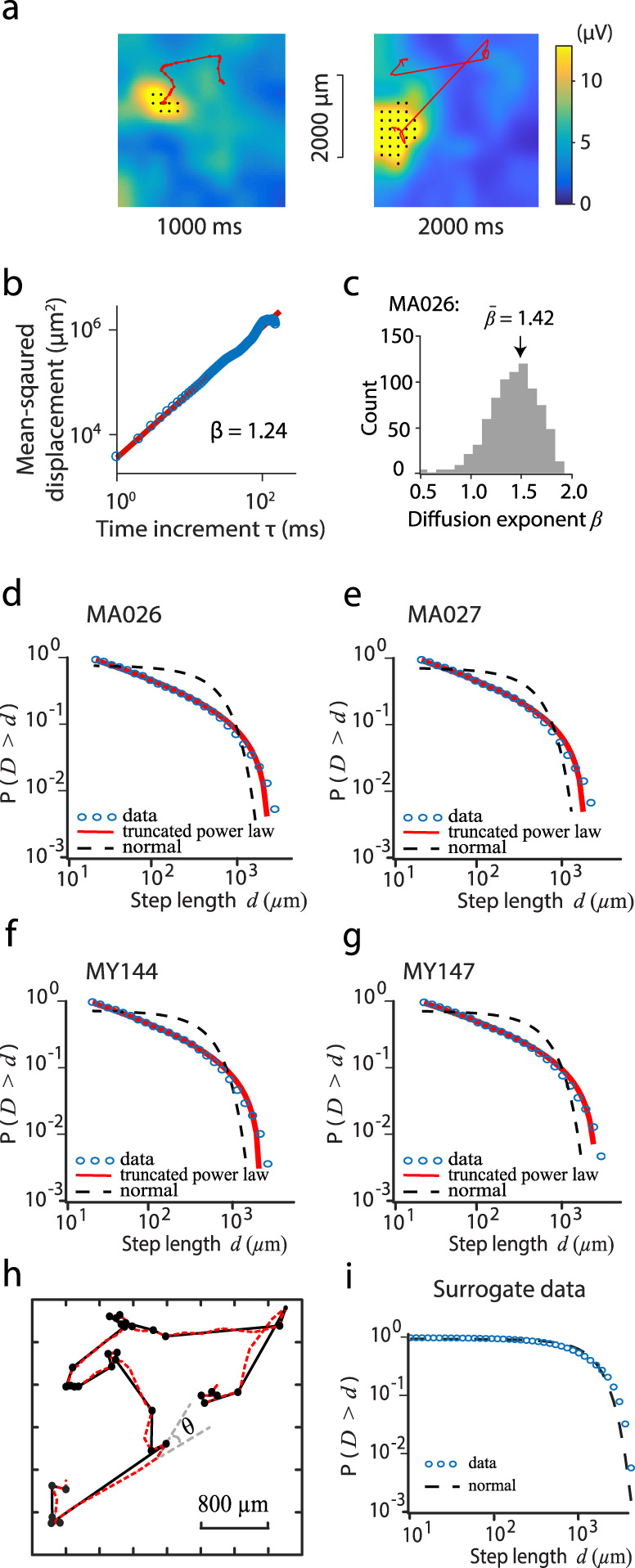
Spatiotemporal dynamics of gamma burst patterns. **a** Snapshots of gamma amplitudes at two timepoints (case MA027; recordings separated by 1 s). Detected bursts are indicated by black dots. Red lines show the trajectories of the center of mass positions of the burst pattern over the previous 100 ms (left) and 300 ms (right). **b** Mean square displacement (MSD) of the trajectory of a typical burst pattern as a function of time increment. Red line indicates a fitted power function of MSD, ${MSD}\left(\tau \right)\propto \,{\tau }^{\beta }$, with diffusion exponent *β* = 1.24. **c** Distribution of diffusion exponent *β* for gamma bursts of animal MA026 (5 min); mean value is 1.42. **d** Complementary cumulative probability distribution (CCPD) of the step lengths (1D model) of MA026 (blue dots). Red line indicates a fitted truncated power distribution with $\lambda$ = 1.32. For comparison, a normal distribution (black dashed line) with mean ${\rm{\mu }}\,=\,0.31{\times 10}^{3}$ and standard deviation $\sigma$ = $0.52{\times 10}^{3}$ is shown. **e** As **d**, case MA027, $\lambda$ = 1.33, ${\rm{\mu }}$ = $0.26{\times 10}^{3}$, $\sigma$ = $0.43{\times 10}^{3}$. **f** As **e**, case MY144, $\lambda$ = 1.33, ${\rm{\mu }}$ = $0.29{\times 10}^{3}$, $\sigma$ = $0.48{\times 10}^{3}$ **g** As **f**, case MY147, $\lambda$ = 1.30, ${\rm{\mu }}$ = $0.32{\times 10}^{3}$, $\sigma$ = $0.52{\times 10}^{3}$ is shown. **h** The angle model used to extract step length from traces ($\theta ={40}^{^\circ }$). Red dashed lines show the trajectories of the center of mass positions of a gamma burst pattern. An example change of angle $\theta$ is shown. The step lengths (black lines) are calculated as the distance between turning points (black dots) where the angle $\theta$ is >${40}^{^\circ }$. **i** Pattern-based surrogate data, ${\rm{\mu }}$ = $0.78{\times 10}^{3}$, $\sigma$ = $0.60{\times 10}^{3}$.

The gamma bursts observed at one electrode were usually accompanied by similar bursts at nearby electrodes, and we found that the gamma bursts were organized as spatially localized patterns. These localized patterns emerged at seemingly random locations and moved across the recorded area with complex spatiotemporal dynamics; Fig. 3a shows such patterns starting at different timepoints. The localized patterns dwelled near one location for a while and then quickly shifted to another location in an intermittent way. To characterize these movement dynamics, we developed a method to track the gamma bursts (see Methods). At each time moment *t*, we calculated the center of mass (CoM) position, ${{\boldsymbol{X}}}_{t}=\left({x}_{t},{y}_{t}\right)$, of the pattern (see Methods). The positions of the CoM at sequential timepoints provide the movement trajectory of a burst pattern (two examples are shown in Fig. 3a). These movement trajectories show superdiffusive dynamics and heavy-tailed, power-law distributions of step lengths, which are two key properties of Lévy walks.

### Superdiffusive dynamics of gamma bursts

To determine whether these trajectories of gamma burst patterns were consistent with the superdiffusive property of Lévy walks, we calculated the mean-squared displacement (MSD), $r\left(\tau \right)=\left\langle {\left|{{\boldsymbol{X}}}_{{\boldsymbol{t}}{\boldsymbol{+}}{\boldsymbol{\tau }}}{\boldsymbol{-}}{{\boldsymbol{X}}}_{{\boldsymbol{t}}}\right|}^{2}\right\rangle$, as a function of time increment $\tau$. The MSD was linear on a log-log scale (Fig. 3b), indicating that that MSD is a power function of time increment *τ*, such that $r\left(\tau \right)\propto \,{\tau }^{\beta }$. Brownian motion is characterized by MSD with *β* = 1; *β* > 1 indicates a superdiffusive process and *β* < 1 indicates a subdiffusive process^20^. We estimated the value of *β* by minimizing the square error between the observations and this model using a Levenberg–Marquardt algorithm (see Methods); 2648 trajectories analyzed in all 4 animals showed a good fit to the model with the residuals < 0.1. The typical trajectory shown in Fig. 3b is a superdiffusive process with *β* = 1.24. The gamma bursts exhibited variable propagation dynamics: some patterns were very mobile while others were more stationary, with the diffusion exponent *β* ranging from 0.3 to 1.9 for all well-fit trajectories (for example, case MA026 in Fig. 3c). Most trajectories appeared superdiffusive: the mean diffusion exponent was $\bar{\beta }=1.42$ (s.d = 0.24, *n* = 773, case MA026). The diffusion exponents for other animals and the aggregated data showed similar distributions with the mean diffusion exponents > 1.0 as well (Supplementary Fig. S1). This superdiffusion suggests that long-range correlations underlie the seemingly random motion of gamma burst patterns^20^.

### Distributions of movement step lengths

We found that the step lengths that form the trajectories of gamma bursts were also consistent with Lévy walk. Step length was defined as the distance travelled between two points that were preceded and followed by either a pause or a change in direction^3,21,22^. We used two methods to detect a change in movement direction: a one-dimensional (1D) model^22–24^ and an angle model^3,21^. As in Rhee et al.^3^, we then characterized the distribution of step length with a complementary cumulative probability distribution (CCPD), which is better than a simple probability distribution at revealing the tails of distributions. In the 1D model, we projected the 2D trajectories onto 1D and defined a turning point as a reversal of movement direction^22,24^. The 1D method is unbiased because it is not dependent on choice of criterion turning angle, while preserving the distribution properties of the original 2D trajectory^24^. We found that the resultant CCPDs of the step lengths of all four animals showed heavy tails (Fig. 3d–g, and the aggregated data shown in Supplementary Fig. S2). In the angle model, we identified turning points as sequential time steps when the change in movement angle was larger than a criterion turning angle, *θ* (Fig. 3h). We varied $\theta$ in ${20}^{^\circ }$ increments from ${20}^{^\circ }$ to ${120}^{^\circ }$ and found that the angle method yielded the similar heavy-tailed distributions as in the 1D model (Supplementary Fig. S3).

The measured trajectories of the gamma burst patterns are naturally bounded by the recording area, which restricts the distribution of movement step-lengths, such that only truncated Lévy walks are experimentally plausible. We therefore tested the CCPD of movement step lengths against a truncated Lévy distribution (that is, a truncated power-law model). We first used Maximum Likelihood Estimation (MLE) to fit five models to the data, including a truncated power-law, exponential, normal, log-normal (a heavy-tailed distribution) and gamma functions (these functions are detailed in Table 1). We then used Akaike Information Criteria weights (AIC) for model comparison and selection^3,25^ (see Methods). We examined distributions of step lengths for individual animals and calculated the AIC weights; Table S1 shows the AIC weights in the 1D model and the angle model with different parameters. In all cases, the truncated power-law distribution had the largest AIC weight among the tested distributions, indicating that it provided the best characterization of the step-length distributions. The exponents of the truncated power-law distribution were all in the Lévy range $1 < \lambda \le 3$, with $\lambda$ = 1.32, 1.33, 1.33 and 1.30 for MA026, MA027, MY144 and MY147, respectively ($\bar{\lambda }$ = 1.32, s.d. = 0.01, *n* = 4; 1D model. Exponents based on the angle model can be found in Supplementary Table S1). The explanatory power of the truncated Lévy distribution was not sensitive to the threshold used for gamma burst pattern detection (1.5 s.d. to 2.5 s.d.) (Supplementary Fig. S4), the minimal step length, or the spatial filtering (Supplementary Note, Fig. S5). These results therefore indicate that the movements of gamma burst patterns were consistent with Lévy walks. We also calculated the movement speeds of the gamma patterns and found that they were very variable with a broad distribution. The average speed was 0.376 m/s (s.d. = 0.309, n = 2.4 × 10^6^ movements), consistent with the propagation speed of gamma patterns found in the visual cortex of rabbit^26^.

**Table 1:** Tested probability density functions.

Model name	Probability density function
Truncated power law	$(-\lambda +1)({b}^{-\lambda +1}-{a}^{-\lambda +1})^{-1}{x}^{-\lambda }$ $(a\le x\le b)$^81^
Exponential	$\frac{1}{\lambda }{e}^{\frac{-x}{\lambda }}$
Normal	$\frac{1}{\lambda \sqrt{2\pi }}{e}^{\frac{-{(x-\mu )}^{2}}{2{\lambda }^{2}}}$
Log-normal	$\frac{1}{x\lambda \sqrt{2\pi }}{e}^{\frac{-{({\rm{ln}}(x)-\mu )}^{2}}{2{\lambda }^{2}}}$ ($x \;> \; 0$)
Gamma	$\frac{1}{{b}^{a}\Gamma (a)}{x}^{a-1}{e}^{\frac{-x}{b}}$ where $\Gamma (a)$ is the Gamma function

We performed parallel analyses on surrogate datasets. One set was generated by randomizing the phases of the entire LFP recordings in the Fourier domain—the Fourier amplitudes were retained to ensure the same autocorrelation as the original data^27^. The same analysis from the raw data to the gamma burst patterns was applied. The numbers of bursts at each electrode were similar in the experimental data (mean: 1317 over all electrodes and all animals, s.d.: 177, *n* = 4 animals) and the surrogate data (mean: 612, s.d.: 19, *n* = 4 animals). However, propagating burst patterns were rarely observed in the surrogate data (${n}_{p}$ < 10 in all recordings), where ${n}_{p}$ is the number of patterns, whereas propagating bursts were common in the original data (mean: 1785, s.d.: 190, *n* = 4 animals, *p* *<* ${10}^{-3}$). This result rules out the possibility that the propagating gamma burst patterns are random events. The second surrogate analysis was done after the gamma patterns were detected. We shuffled the CoM position within each detected trajectory (mean duration: 79.13 ms, s.d.: 107.31 ms, *n* = 7027 for four animals) and then applied the procedure described above to study the step length distribution; the distribution of step length of the surrogate data follows a normal distribution (Fig. 3i). These results indicate that the Lévy walk dynamics is an inherent property of gamma burst patterns.

### Gamma burst patterns in model circuits

**Fig. 4:**
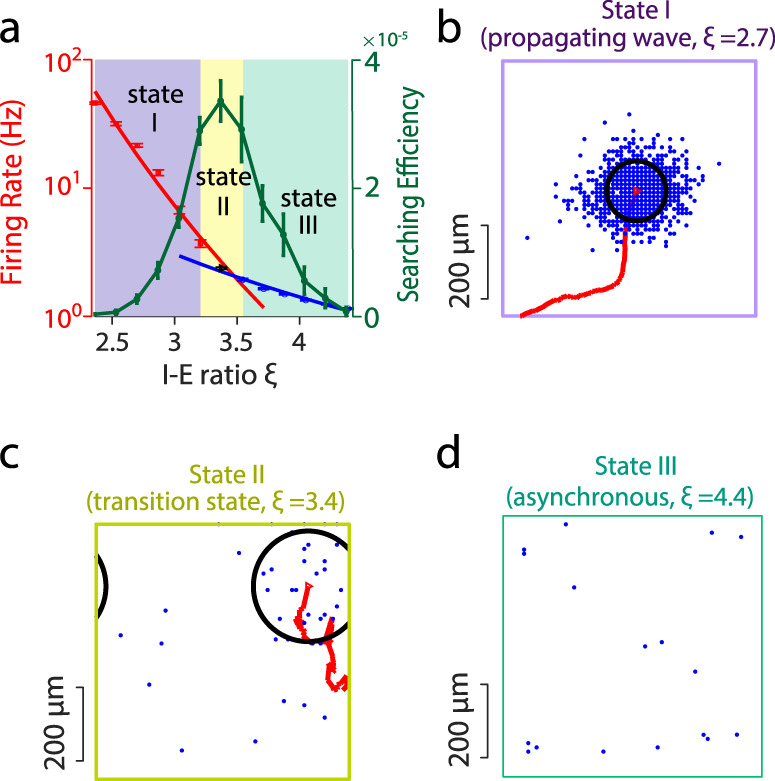
Emergent network activity in a neural circuit model. **a** Mean firing rate of the population of excitatory neurons in the model shows a phase transition around an I-E ratio of 3.4. The red and blue lines are two power functions fitted to the data points marked by red squares and blue circles, respectively. The black dot denotes the crossing point of the two power-law functions. The green line shows the change of the searching efficiency with different I-E ratios. Error bars show SEM. Three distinct states emerging from the circuit are marked by different colors; State III corresponds to the asynchronous state, State I exhibits localized propagating wave patterns and State II is the transition region. **b**–**d** Snapshots of neural spiking patterns emerging from the circuit model with different I-E ratio values. Blue dots denote spikes emitted by excitatory neurons during a 5 ms period. Black circles in (**b**) and (**c**) show one standard deviation of the 2D normal fitted firing rate. Red curves show trajectory of the pattern in the previous 100 ms. For State I in (**b**), the spiking patterns propagate smoothly. For State II in (**c**), the pattern appears intermittently and exhibits variable propagation trajectories. For State III (the asynchronous state) in (**d**), no patterns are formed.

To understand what circuit mechanisms could underlie the Lévy walk dynamics of gamma burst patterns, we implemented a biophysically plausible, spiking circuit model with excitatory and inhibitory neurons that capture the known anatomy and physiological of cortical circuits^15^. It incorporates distance-dependent synaptic connectivity and balanced excitation and inhibition^15^ (Methods). The model exhibits a rich repertoire of dynamic activity, ranging from asynchronous to propagating wave states (Fig. 4). If the inhibition-to-excitation (I-E) ratio $\xi$ is small ($\xi$ < ${\xi }_{c};$ ${\xi }_{c}$ = 3.4), localized propagating waves emerge and propagate across the neural circuit with relatively smooth and regular moving trajectories (Fig. 4b). On the other hand, if the I-E ratio is large ($\xi$ > ${\xi }_{c}$), the circuit exhibits an asynchronous state without any structured patterns in spiking activity (Fig. 4d). When the circuit is close to the transition region between the asynchronous state and the wave state ($\xi \approx {\xi }_{c}$), localized spiking activity patterns emerge intermittently and exhibit complex spatiotemporal dynamics (Fig. 4c) consistent with the Lévy walk dynamics of gamma bursts found in our experimental data.

**Fig. 5:**
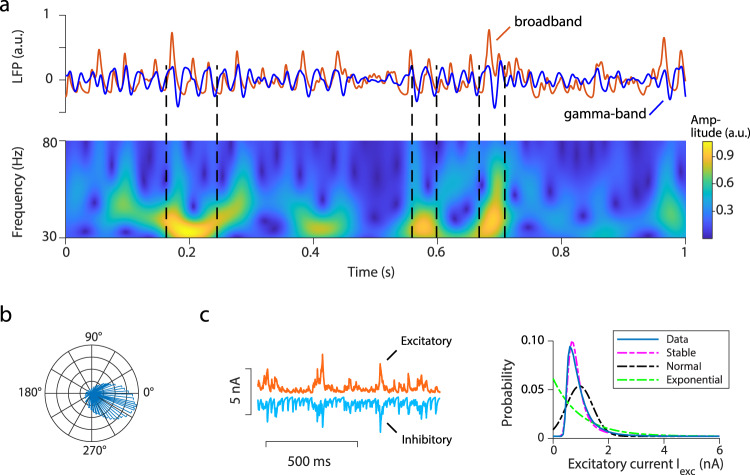
Properties of gamma bursts in a neural circuit model. **a** Raw (red) and gamma-band filtered (30–80 Hz, blue) LFP time series. Lower panel shows the corresponding Morlet wavelet spectrogram of the gamma-band LFP time series. Black dashed lines denote gamma bursts. **b** Distribution of spikes of model excitatory neurons at different phases of gamma bursts, indicating phase-locking to gamma bursts. **c** Left: time series of excitatory and inhibitory inputs received by one model neuron over a period of 1 s. Note correlated and proportional fluctuations in excitation and inhibition. Right: probability distribution of excitatory currents received by a single model neuron (blue). Magenta dashed line denotes a fitted Lévy stable function ($\alpha$ = 1.216) of the distribution. Normal distribution (black dashed line) and exponential function (green dashed line) are shown to be  inadequate to describe the distribution.

We derived LFP signals from our spiking neural circuit model (see Methods). The model LFP trace exhibits transient epochs of gamma oscillations with varying duration (top panel, Fig. 5a); such gamma bursts are also present in the model spectrogram (bottom panel, Fig. 5a). Individual neurons in the model fire variably with mean coefficient of variation of interspike intervals ~0.96; these variable spikes are phase locked to gamma bursts (Fig. 5b), consistent with empirical observations^9,14^. Further, the amplitudes of both excitatory and inhibitory postsynaptic currents onto individual neurons exhibit large cycle-to-cycle fluctuations (Fig. 5c, left). Despite these fluctuations, excitation and inhibition in the circuit model are tightly balanced (Fig. 5c, left), as measured in experimental studies^28^. The I-E ratio is ~3.4; it is interesting to note that this value and the presence of large fluctuations are quantitatively consistent with experimental studies of gamma oscillations^29^. The fluctuations form a skewed distribution with a heavy-tail (Fig. 5c, right). We used the MLE method to fit the Lévy stable, normal and exponential functions to the distribution. The probability density function of Lévy stable distribution is $P\left(x\right) \sim \alpha \gamma {c}_{\alpha }\left(1+{{\rm{\alpha }}}_{s}\right){x}^{-\left(\alpha +1\right)}$, where $\gamma$ is the scale parameter, ${c}_{\alpha }$ = sin(πα/2)Γ(α)/π, Γ is the Gamma function, ${{\rm{\alpha }}}_{s}$ is the skewness parameter and $\alpha$ is the stability parameter with the range 0 < $\alpha$ < 2^30^. The AIC weights showed that the Lévy stable distribution provided the best characterization. The stability parameter $\alpha$ of the fitted Lévy stable distribution is 1.216 (confidence interval $\alpha \in$ [1.214, 1.218]). Such large fluctuations only occur in the transition regime of the circuit. In the asynchronous state, the synaptic inputs stabilize and have Gaussian-like statistics. In the propagating wave state, the inputs become semi-periodic and regular. The synaptic fluctuations of our model resemble the large fluctuations typically observed in complex physical systems near phase transitions of different states^31^.

Consistent with empirical observations^29^, fluctuations in amplitudes of both excitatory and inhibitory currents in our model are correlated with the amplitudes of LFP gamma oscillation on a cycle-by-cycle basis (excitatory: *r* = 0.43 ± 0.27, *p* *<* 10^−3^; inhibitory: *r* = 0.55 ± 0.15, *p* *<* 10^−3^). The amplitude of each gamma oscillation cycle is strongly correlated with the latency to the subsequent cycle (*r* = 0.76 ± 0.01, *p* *<* 10^−3^) with higher amplitude leading to longer latency of the next cycle; this is a characteristic feature of gamma oscillations in rat hippocampus^29^ and awake macaque area V1^32^. The duration of each gamma cycle is also inversely correlated with the average spike rate (*r* = −0.54 ± 0.01, *p* *<* 10^−3^), as found by Spyropoulos et al.^32^.

**Fig. 6:**
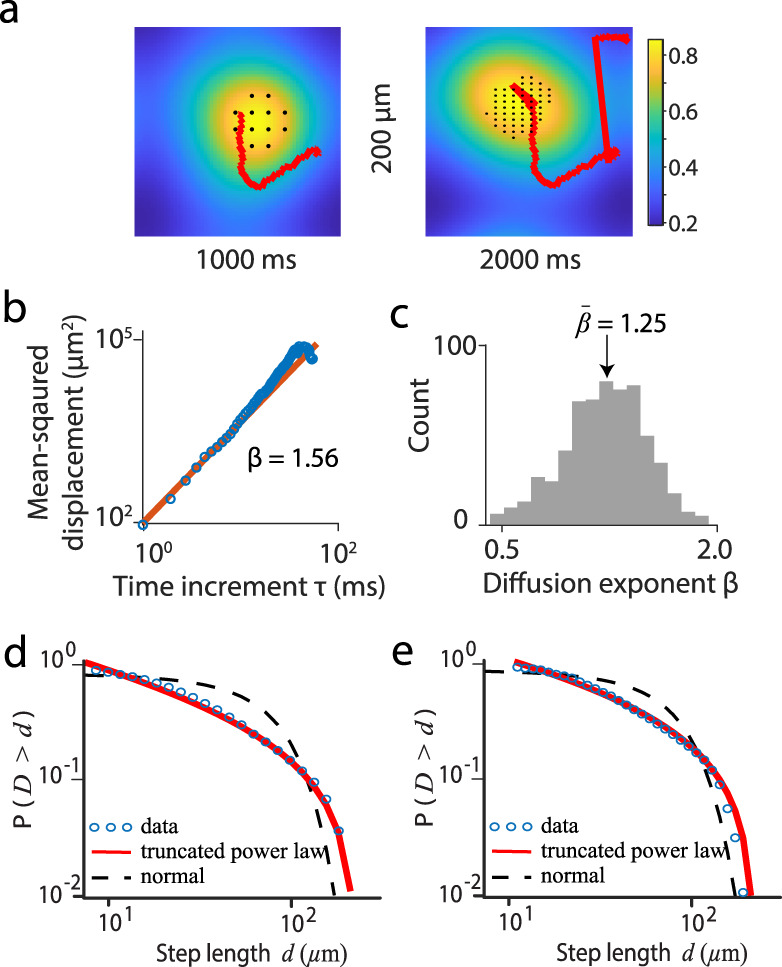
Spatiotemporal dynamics of gamma patterns in the circuit model. **a** Snapshots of gamma-band LFP amplitudes at two timepoints. Black dots show the positions of gamma bursts in the model circuit. Amplitude patterns are overlaid with representative trajectories (red lines) of the center of mass of the burst patterns over the previous 30 ms (left) and 270 ms (right), respectively. **b** Mean square displacement (MSD) of a typical spiking pattern as a function of time increment. Red line shows a fitted power function of MSD, ${MSD}\left(\tau \right)\propto \,{\tau }^{\beta }$, with the diffusion exponent *β* = 1.56. **c** Distribution of diffusion exponent *β* for spiking pattern in simulation data (1000s); mean value is 1.25. **d** CCPD of the step lengths (1D model) of the activity pattern in the transition state (State II) (blue circles). Red line indicates a fitted truncated power distribution with $\lambda$ = 1.32. For comparison, a normal distribution (black dashed line) with mean ${\rm{\mu }}$ = $0.59{\times 10}^{2}$ and standard deviation $\sigma$ = $0.69{\times 10}^{2}$ is shown. **e** as **d** for angle model: ($\theta ={40}^{^\circ }$), $\lambda$ = 1.13, ${\rm{\mu }}$ = $0.10{\times 10}^{3}$, $\sigma$ = $0.95{\times 10}^{2}$.

We next tracked and analyzed the trajectories of the localized activity patterns produced by the model, using the same methods we used to analyze the experimental data (Fig. 6a). Figure 6b shows the MSD of a typical burst pattern, which is a power function of time increment $\tau$, $d\left(\tau \right)\propto \,{\tau }^{\beta }$, with *β* = 1.56. The distribution of diffusion exponents in the model is likewise comparable to that found in our experimental data (Fig. 6c), with the diffusion exponent *β* ranging from 0.48 to 1.98 (mean $\bar{\beta }$ = 1.25). We detected the turning points of movement trajectories as described above and used these points to define the distributions of step lengths, again characterizing the CCPDs using MLE to derive AIC weights and determine the best model (see Supplementary Table S2). We found that the CCPDs for both the 1D and angle methods were best characterized by a truncated power law distribution with exponents in the Lévy range $1 < \lambda \le 3$ (Fig. 6d and Fig. 6e), as in the experimental data.

Shifting the model network away from the dynamic regime and further into the asynchronous regime led to a loss of localized activity patterns (Fig. 4d). By contrast, shifting the network further into the wave regime resulted in a regular propagation of activity patterns (the red curve in Fig. 4b indicates a smooth trajectory) lacking the spatiotemporal dynamics characteristic of the transition regime. It is also interesting to note that in the critical transition regime, the pattern propagation speed was highly variable (Supplementary Fig. S6) as found in our data. However, the mean of the propagation speeds was 11.2 μm/ms, which was much smaller than that found in our data. This discrepancy is likely because the total number of neurons of our network is much smaller than real neural circuits. In the excitation-dominated state of our model, the localized wave patterns propagated more slowly (~6.82 μm/ms) than the patterns in the critical transition regime and did not exhibit significant variability.

### Metabolic efficiency of activity patterns sampling cortical space

To test for a functional advantage of the activity pattern with Lévy walks in the transition regime, we used the neural circuit model to calculate a spike energy budget while the pattern moves over the cortical surface. To quantify the sampling capacity of the spiking pattern, we uniformly divided the 2-dimensional network into ${N}_{t}=100$ small areas (i.e., squares) and calculated the number of different squares (${N}_{s}$) visited by the CoM within 3 s. The sampling rate is ${S}_{r}=\frac{{N}_{s}}{{N}_{t}}$.

To calculate neural energy expenditure we used the method of Lévy and Baxter^33^: in each time window $\triangle \tau =50$ ms, each neuron costs *r* energy units due to leak currents and each spike costs one extra unit of energy, so the energy expenditure is:

1 $$E=m+{nr}$$

where *m* is the number of spikes during the time interval, *n* is the total number of neurons in the network. Based on Eq. (1), we then obtained the total energy expenditures ${E}_{{total}}$. The sampling efficiency of the activity pattern is defined as the sampling rate ${S}_{r}$ divided by the total energy expenditure, $\eta =\frac{{S}_{r}}{{E}_{{total}}}$. The searching efficiency indicates that the larger the value $\eta$, the more metabolically efficient it is for the pattern to sample the 2D cortical space. In our calculation we used *r* = 0.01$,\triangle t=50$ ms and ${N}_{t}=100$ to calculate the sampling efficiency; other choices near these values yielded similar results.

In our circuit model, we found that as the I-E ratio ($\xi$) decreases, the sampling rate ${S}_{r}$ increases (Supplementary Fig. S7) and that the propagating wave regime (State I) provides higher overall$\,{S}_{r}$ than other regimes. The energy expenditure ${E}_{{total}}$ also increases as $\xi$ decreases (Supplementary Fig. S7). However, as shown in Fig. 4a (green line) the sampling efficiency $\eta$ ($\eta =\frac{{S}_{r}}{{E}_{{total}}}$), which is the trade-off between the sampling rate and the energy expenditure, is maximal when the network is in the critical transition region (State II, where the I-E ratio is ~3.4). The sampling efficiency in State II is significantly larger than the values of $\eta$ in either State I or State III (*p* *<* ${10}^{-3}$, Kolmogorov–Smirnov test). This result thus indicates that the activity pattern with Lévy walk dynamics are an energy-efficient way to dynamically sample the cortical space.

## Discussion

We find that the spatiotemporal organization of gamma bursts in primate cortex can be characterized as Lévy walks. Our circuit model explains how the Lévy walk dynamics can arise from intrinsic, near-critical transitions between cortical states. Thus, gamma bursts exhibit more complex spatiotemporal dynamics than expected by conventional views of stable steady states^34–37^ or filtered Gaussian noise^38,39^. In the following we first relate the Lévy walk dynamics revealed here to previous studies of gamma bursts, then consider the origin of these dynamics in synaptic activity as illustrated by our circuit model. Finally, we consider implications for cortical function.

### Spatial and temporal properties of gamma bursts

The Lévy walk property means that localized gamma burst patterns hover around one location for a while and then move or jump to another location in an intermittent manner. These spatiotemporal dynamics can reconcile and extend some previous findings on gamma oscillations. For instance, spatially localized gamma oscillations were reported by Freeman and Barrie^26^ and Sirota et al.^40^ but their spatiotemporal evolution was not studied. The Lévy walk characteristic of gamma bursts means they occasionally exhibit long propagation trajectories; this property would yield a wave-like propagation as previously described^41–43^. At the same time, the intermittent dwelling of gamma bursts gives rise to gamma synchrony (correlation) of neurons in the local cortical area^34,35^; we show here that such gamma synchrony is localized in space but is transient in time.

Other work has shown that the magnitude of gamma oscillation is modulated by slower oscillations such as theta oscillation^44,45^. Our findings imply that such cross-frequency coupling would also exhibit complex spatiotemporal dynamics. If propagating patterns at different frequencies are coupled across multiple spatial and temporal scales, the resulting cascades of pattern interactions could be comparable to cascaded interactions in coherent structures such as vortices in turbulent fluids^46^.

Overall, our results show that the simple concept of temporal correlation, which has guided our understanding of gamma oscillations during the past two decades^34,47^, can usefully be expanded to take spatial variables into account; such expansion can provide a new perspective for understanding gamma oscillations.

### Modeling Lévy walk dynamics of gamma bursts

Our modeling indicates that Lévy walk dynamics of gamma burst patterns depends on critical transitions between asynchronous states and propagating wave states. Lévy walks arising from criticality have been mainly found in models of low-dimensional dynamical systems. For example, one recent study^48^ showed Lévy walks can by generated by coupled chaotic oscillators near the transition between synchronous and asynchronous states. In ref. ^49^, bursty time-series of coupled Lotka–Volterra (L–V) models and spike potentials recorded from rat hippocampus were used to generate superdiffusive Lévy processes. Complex neural dynamics have been characterized as critical avalanches^50^, but it is unclear whether these avalanches could be organized as localized activity patterns with Lévy movements in space. In contrast, our model is neural circuit-based and incorporates properties such as distance-dependent synaptic coupling^15^, synchronized excitatory and inhibitory synaptic fluctuations (as measured empirically by Atallah and Scanziani^29^), and localized propagating patterns.

In classical neural circuit models, in contrast to our model, neurons fire in regular fashion, so these classical models are unable to capture variable spike rates and non-stationary bursts^36,37,51^. Population gamma oscillation and variable spike rates can be reconciled in the sparse synchrony regime of randomly coupled circuits^47,52^. But synaptic inputs in sparse synchrony models are characterized as Gaussian noise^53^, which is incompatible with the heavy-tailed distributions that we observe experimentally and in our circuit model. Heavy-tailed, non-Gaussian properties often emerge from complex non-equilibrium systems, suggesting that methods for analyzing such complex systems (for example, fractional Fokker–Plank formalisms^54^) could be a promising direction to pursue for formal analysis of the Lévy walk dynamics.

Gamma bursts have been widely observed in many brain regions^9–14,26,55^. The spatiotemporal dynamics of gamma bursts that we here have analyzed in the cerebral cortex may therefore be applicable to other brain areas. High-frequency oscillations at other frequencies bands such as beta (13–30 Hz)^56^ and sharp wave ripples (140–220 Hz)^57^ also exhibit transient bursts, and the presence of jump-like behavior of sharp wave ripple patterns has been reported in hippocampus^58^. Our preliminary analysis of delta– and theta-band activity indicates activity in these frequency bands does not show clear Lévy walk dynamics but a full exploration of this question is beyond the scope of the present study.

### Relevance to brain function

What could be the relevance of our results for understanding information processing in the brain? First, it is natural to ask whether the results we found here under anesthesia would apply to the waking state. In the waking state, gamma oscillations are associated with functions including sensory processing^59^, cognition, memory^35,44,60^ and attention^61,62^. In the anesthetized state, and at rest in absence of patterned sensory stimulus (resting state: Fox and Raichle^63^) the amplitude and coherence of gamma oscillations is decreased but their dynamic properties are otherwise preserved^9,64^. Likewise the main change in subcortical visual evoked spike activity under sufentanil anesthesia is reduced response amplitude without other changes in receptive field properties^65^. Finally, resting-state fluctuations (in terms of up and down changes over time) in blood-oxygen-level-dependent fMRI signals are preserved at anesthetic levels that produce profound loss of consciousness (0.8–1.5% isoflurane)^66^. These examples of preserved elementary brain functions under anesthesia predict that basic properties of Lévy walk dynamics in gamma bursts will be preserved in the waking state. Such experiments are beyond the scope of the present study, but it would be straightforward to apply our circuit model to recordings from waking brains.

Lévy walk movements have been observed of Drosophila larva neural circuits^8^ and in other biological systems and complex physical systems^1,6,20^. For instance, Lévy walks are essential for optimally transporting energy in turbulent fluids^67^, for animals to optimally search for spatially distributed food^7^, and for *lymphocytus* T-cells to efficiently find target pathogens in brain explants^4^. In the context of normal brain function we propose here two hypotheses, which are not mutually exclusive, as follows.

Our first hypothesis relates to the fact that gamma bursts have been widely observed in attentional and cognitive tasks. The Lévy walk dynamics of gamma bursts may help an attentional “spotlight” to focus on one location for a while and then switch/jump to another location in an intermittent manner. Relatedly, the scale-free property of Lévy walks could provide an efficient mechanism for sampling natural environments, which are inherently scale-free^68,69^. It has been found that saccadic shifts when viewing natural scenes follow Lévy motion^70^. Attention and saccadic eye movement are closely related, as visual spatial attention determines the end point of saccades^71^. Taken together, these observations suggest the Lévy motion property of gamma bursts may improve the efficiency of attention sampling. This hypothesis could be tested by neural population decoding^72^, and comparing the statistical properties of spatial attention to predictions of Gaussian and Lévy walk dynamics. Based on our results (Fig. 3, Fig. 6) these predictions would diverge for temporal scales above 100 ms and spatial scales represented by 100–1000 µm on the surface of area MT, at least in marmoset monkeys. The question whether the same scale factors hold in larger brains or unanaesthetized brains remains to be tested.

Our second hypothesis relates to the metabolic expense of spiking activity in the cerebral cortex^16,17^ and the associated evidence for sparse spiking as a basis for information processing in the brain^73–75^. Our modeling (Fig. 4) showed that activity patterns with Lévy walks dynamics are a metabolically efficient way to visit spatially distributed locations on the cortical sheet; that is, the ratio of the locations visited to the energy consumed is maximal in the transition regime where Lévy walks dynamics emerge. In this context, the superdiffusion dynamics of gamma bursts would enable widely separated regions of cortex to be activated within short time steps. By analogy, cortical activity patterns could be seen as efficient “foraging” for spikes which are separated in space but close together in time. This hypothesis could be tested by comparing Gaussian to Lévy walk statistics of gamma burst activity, at the millisecond timescales relevant to spike-dependent plasticity^76^ and related phenomena.

## Materials and methods

Multielectrode array (10 × 10 electrodes, 1.5 mm length, electrode spacing 400 µm, Blackrock Microsystems) recordings were made from the MT cortical area of four adult male marmosets (*Callithrix jacchus*). Procedures conformed to the Australian National Health and Medical Research Council Code of Practice for the use and care of animals, and were approved by institutional committee at the University of Sydney. Details of the preparation are given elsewhere^77^. Anesthesia and analgesia were maintained by intravenous sufentanil infusion (6–30 $\mu g\cdot{{kg}}^{-1}{h}^{-1}$) and inspired 70:30 mix of ${N}_{2}O$ and carbogen. The eyes were held open with micro-retractors and protected with oxygen-permeable contact lenses. Refraction was optimized (with optometric trial lenses) by maximizing the response of the first recorded units to high spatial frequency drifting gratings. Stimuli were viewed through natural pupil, which typically had diameter close to 2 mm. The animal viewed a uniform gray field presented on a cathode-ray-tube monitor (Sony G500, refreshed at 100 Hz, viewing distance 45 cm, mean luminance 45–55 cd m^−2^). Recording surface insertion depth was targeted to 1 mm. The LFP sampling frequency was 1024 Hz. Recording duration ranged from 5 to 25 min in total: the first 5 min of recording were analyzed for each animal (*n* = 4). The identification numbers of the four animals are MA026, MA027, MY144 and MY147. The four corner electrodes were excluded for all the animals. For animal MY144, electrodes [3, 8] and [5, 4] were excluded due to bad signal quality. The missing channels were interpolated by a Gaussian filter with filter width of 3 electrodes and standard deviation of 0.6.

### Detection of gamma burst patterns

Electric line noise at 50 Hz was removed by applying a second-order Butterworth band-stop filter to the raw LFP signals. Zero-phase forward and reverse filters were used to preserve features in the filtered waveform. Following Lundqvist et al.^12^, bandpass filtering and/or wavelet analysis were applied to calculate gamma-band signal amplitude; the methods yielded similar results and comparable burst extraction outcomes (Fig. 2d). For bandpass filtering analysis, a 4th order Butterworth bandpass filter was applied to the LFP signals, the first and final 1 s of the record were discarded to reduce boundary effects, and amplitude was extracted using the Hilbert transform. For wavelet analysis, the Morse wavelet transform was applied to the LFPs with symmetry parameter 3 and the time-bandwidth product^12^.

To characterize gamma burst patterns, a smoothing Gaussian filter (s.d. 0.6) was applied to the resized (factor 4, bi-cubic interpolation) electrode matrix of gamma amplitudes. Other smoothing and resizing parameters yielded comparable results. Neural data are then represented as *z(x, y, t); z* is the recorded signal at the position *(x, y)* at time moment *t*. Following Lundqvist et al.^12^, a burst event at (*x, y*) is defined where the gamma amplitude exceeds (by 2.5 s.d.) the trial mean value; other threshold values yielded similar results. We defined burst structures as a spatial connected set of burst points, with connectedness defined in terms of the six orthogonal neighbours in the Cartesian mesh of the recorded sites (“bwconncomp” routine in MATLAB 2016b; MathWorks, Natick, MA). If the burst structures were connected in the consecutive time framework, these structures were identified as gamma burst patterns. Burst patterns with duration >60 ms and spatial extent (maximum in *x* or *y* direction >1.2 mm) were analyzed. 7027 patterns were detected for the four animals (1866, 1595, 1615, and 195l patterns for the animal MA026, MA027, MY144 and MY147, respectively).

### Analysis of step lengths

To study the trajectory of the gamma burst patterns, the centre of mass ${{\boldsymbol{X}}}_{t}=\left({x}_{t},{y}_{t}\right)$ of the detected patterns at each time-step was calculated as the instantaneous location of the patterns,

2 $${{\boldsymbol{X}}}_{{\boldsymbol{t}}}=\frac{\mathop{\sum }\limits_{i}^{{N}_{t}}{{\boldsymbol{X}}}_{{\boldsymbol{t}}}^{{\boldsymbol{i}}}\left({x}_{t},{y}_{t}\right)}{{N}_{t}}$$

where ${{\boldsymbol{X}}}_{{\boldsymbol{t}}}^{{\boldsymbol{i}}}\,\left({x}_{t},{y}_{t}\right)$ is the location of the *i*-th burst within a detected gamma burst pattern at time *t* and ${N}_{t}$ is the total number of bursts within the pattern. To demonstrate the robustness of our results, we also tested our results by defining the amplitude weighted centre of mass (${{\boldsymbol{X}}}_{{\boldsymbol{t}}}^{{\boldsymbol{w}}}=\frac{\mathop{\sum }\limits_{i}^{{N}_{t}}{Z}_{i}\left(x,y,t\right){{\boldsymbol{X}}}_{{\boldsymbol{t}}}^{{\boldsymbol{i}}}\left({x}_{t},{y}_{t}\right)}{\mathop{\sum }\limits_{i}^{{N}_{t}}{Z}_{i}\left(x,y,t\right)\,}$). Both definitions yielded similar results.

Next, the time series of locations was converted into a series of movement steps. Here, two methods were used to define movement steps. The first method used is the 1D model. In this model, the 2D pattern trajectories were projected to the *x* direction. Turns were identified as a reversal in direction in the 1D movement. Step length was then defined as the distance between the turns. The second is the 2D turning angle model. Given two points at ${\boldsymbol{X}}\left(t\right)$ and ${\boldsymbol{X}}\left(t+1\right)$, we calculated the distance between these two points and considered this distance a step if the angle formed by ${\boldsymbol{X}}\left(t\right)$, ${\boldsymbol{X}}\left(t+1\right)$ and the next point ${\boldsymbol{X}}\left(t+2\right)$ is greater than the characteristic angle (*θ* as shown in Fig. 3h). If the condition was not met, we calculated the distance between ${\boldsymbol{X}}\left(t\right)$ and ${\boldsymbol{X}}\left(t+2\right)$ and determined whether these points met our criteria. This process was continued until a step length was found that met the criteria described above. In addition, the distance between the location where one pattern disappears and a new pattern emerges was also considered as a step. Different turning angles were used to interrogate the 2D turning angle models (Supplementary Fig. S3).

### Maximum likelihood estimation (MLE) and model selection

We used the MLE method^78^ to fit truncated power law, exponential, normal, log-normal and gamma distributions. Briefly, MLE finds estimated parameter that maximizes the likelihood function in all the models. The probability density functions for all the models are shown in Table 1. The step lengths smaller than 20 µm were firstly discarded as they are below the minimal resolution. The remaining step lengths were used to find the best fit for all the models using MLE respectively. When fitting the truncated power law, an iterative process was used to derive a best fit value for the parameter *a* and *b* (the lower bound and the upper bound of the truncated power law distribution).

After the best fit for all the distributions were found, the log-likelihoods (LLH) for all the distributions were calculated^78^. Then, AIC weights were employed for model selection^3,25^,

3 $${\mathrm{{AIC}}}=-2{{\log }}\left(L\left(\hat{\theta }|{data}\right)\right)+2K$$

where $L\left(\bullet \right)$ is the likelihood function and $K$ is the number of estimable parameters (the value of $\theta$) in the approximating model (probability distribution).

As AIC values contain arbitrary constants and are greatly affected by the sample size, they do not represent an absolute metric and cannot be used directly. The following transformation makes the result an interpretable metric^3^:

4 $${\triangle }_{j}={{{{AIC}}}}_{j}-{{{{AIC}}}}_{{\min }}$$

where ${{AIC}}_{j}$ is the AIC value of the $j$th model and ${{AIC}}_{{\min }}$ is the minimum of different AIC values. The Akaike weights ${w}_{j}$ are useful as the weight of evidence^3^,

5 $${w}_{j}=\frac{{{\exp }}\left(-\frac{{\triangle }_{j}}{2}\right)}{{\sum }_{r=1}^{R}{{\exp }}\left(-\frac{{\triangle }_{r}}{2}\right)}$$

where *R* is the size of a set of the approximating models (distribution). In addition to wAIC, a Vuong test of the truncated power law was used to check if the competing distribution should be rejected.

### Neural circuit model of excitory and inhibitory neurons

Our circuit model was described in Gu et al.^15^. Briefly, ${N}^{E}$ excitatory and ${N}^{I}$ inhibitory model neurons are uniformly located in a 2D square plane and connected by chemical synapses with probability ${p}^{\alpha \beta }\,$(*α* is the post-synaptic neuron population, and *β* is the pre-synaptic population); ${p}^{\alpha \beta }$ is an exponential distance-dependent connection probability function between connected neurons. The subthreshold membrane potential ${V}_{i}^{\alpha }$ of neuron $i$ in population $\alpha$ follows

6 $$C\frac{d{V}_{i}^{\alpha }\left(t\right)}{{dt}}=-{g}_{L}\left[{V}_{i}^{\alpha }\left(t\right)-{V}_{L}\right]+{{I}_{i,K}^{\alpha }\left(t\right)+I}_{i,{rec}}^{\alpha }\left(t\right)+{I}_{i,{ext}}^{\alpha }\left(t\right)$$

where the membrane capacitance $C$ = 0.25 nS, the leak conductance ${g}_{L}$ = 16.7 nS, and ${V}_{L}\,$ = −70 mV is the reversal potential for the leak current. ${I}_{i,K}^{\alpha }\left(t\right)\,$is the potassium current, ${I}_{i,{rec}}^{\alpha }\left(t\right)$ is the recurrent synaptic current received by the neuron and ${I}_{i,{ext}}^{\alpha }\left(t\right)$ is the external current. When the membrane potential reaches the threshold ${V}_{{th}}=-50\;{\rm{mV}}$, a spike is emitted and the membrane potential is reset to the potential ${V}_{{rt}}=-60\;{\rm{mV}}\,$for an absolute refractory period ${\tau }_{f}=4\;{\rm{ms}}$﻿. The potassium current is given by ${I}_{i,K}^{\alpha }\left(t\right)=-{g}_{i,K}^{\alpha }\left(t\right)\left({V}_{i}^{\alpha }\left(t\right)-{V}_{K}\right)$, where ${g}_{i,K}^{\alpha }\left(t\right)$ is the active potassium conductance and ${V}_{K}$ = −85 mV. The dynamics of the potassium conductance are described by

7 $$\frac{d{g}_{i,K}^{\alpha }\left(t\right)}{{dt}}=-\frac{{g}_{i,K}^{\alpha }\left(t\right)}{{\tau }^{K}}+\triangle {g}_{K}\mathop{\sum}\limits_{k}\delta \left(t-{t}_{j,k}^{\alpha }\right)$$

where ${t}_{j,k}^{\alpha }$ is the time of the ${k}^{{th}}$ spike emitted by neuron $i$ from population $\alpha$, $\triangle {g}_{K}$ = 10 nS and ${\tau }^{K}$ = 80 ms. Because spike frequency adaptation has been primarily observed in cortical pyramidal neurons, we only include such adaptation for excitatory neurons in our model.

The recurrent synaptic current$\,{I}_{i,{rec}}^{\alpha }\left(t\right)$ in Eq. (6) is:

8 $${I}_{i,{rec}}^{\alpha }\left(t\right)=-\mathop{\sum}\limits_{\beta }{g}_{i}^{\alpha \beta }\left(t\right)\left({V}_{i}^{\alpha }\left(t\right)-{V}_{{rev}}^{\beta }\right)$$

where ${g}_{i}^{\alpha \beta }\left(t\right)$ is the conductance of the recurrent current from the pre-synaptic population $\beta$. The excitatory and inhibitory reversal potential are ${V}_{{rev}}^{E}=0\;{\rm{mV}}$ and ${V}_{{rev}}^{I}=-80\;{\rm{m}}$V, respectively. The conductance ${g}_{i}^{\alpha \beta }\left(t\right)\,$is given by

9 $${g}_{i}^{\alpha \beta }\left(t\right)=\mathop{\sum }\limits_{j=1}^{{N}^{\beta }}{a}_{{ij}}^{\alpha \beta }{J}_{{ij}}^{\alpha \beta }{s}_{{ij}}^{\alpha \beta }\left(t\right)$$

where connection topology ${a}_{{ij}}^{\alpha \beta }$ and strength ${J}_{{ij}}^{\alpha \beta }$ capture the numbers of connections and coupling weights respectively, as detailed in Gu et al.^15^. The non-dimensional gating ${s}_{{ij}}^{\alpha \beta }\left(t\right)$ describes the synaptic dynamics,

10 $$\frac{{{ds}}_{{ij}}^{\alpha \beta }\left(t\right)}{{dt}}=-\frac{{s}_{{ij}}^{\alpha \beta }\left(t\right)}{{\tau }_{d}^{\beta }}+\mathop{\sum}\limits_{k}{h}^{\beta }(t-{t}_{j,k}^{\beta }-{d}_{{ij}}^{\alpha \beta })(1-{s}_{{ij}}^{\alpha \beta }(t))$$

11 $${h}^{\beta }\left(t\right)=\left\{\begin{array}{ll}1/{\tau }_{r}^{\beta },&{if}0\le t\le {\tau }_{r}^{\beta }\\ 0,&{otherwise}\end{array}\right.$$

where ${\tau }_{d}^{\beta }$ and ${\tau }_{r}^{\beta }$ are decay and rise time constants respectively, ${t}_{j,k}^{\beta }$ is the time point of the ${k}^{{th}}$ spike of neuron $j$ from population $\beta$, and ${d}_{{ij}}^{\alpha \beta }$ is the conduction delay drawn from a uniform distribution between 0 and 4 ms. We implemented a vital neurophysiological feature of mammal primary cortex in our model^15^, that is, there is an identical ratio between the excitatory post-synaptic currents and inhibitory post-synaptic currents with respect to the whole excitatory population. To model this in our circuit, we consider the I-E ratio ${\xi }_{i}={\sum }_{k}^{{K}_{i,{in}}^{{EI}}}{J}_{{ik}}^{{EI}}/{\sum }_{j}{J}_{{ij}}^{{EE}}$, where ${K}_{i,{in}}^{{EI}}\,$denotes the number of connections (in-degree) received by neuron $i$ from the inhibitory population. To equalize the I-E ratio ${\xi }_{i}$ across the neurons to a desired network-wide ratio, that is, $< {\xi }_{i} > =\xi$, the ${J}_{{ik}}^{{EI}}$ values for neuron *i* are sampled from a Gaussian distribution with a mean equal to $\xi {\sum }_{j}{J}_{{ij}}^{{EE}}/{K}_{i,{in}}^{{EI}}$ and a standard deviation that is 25% of the mean. The I-E ratio $\xi$ is varied as a system parameter to explore the spatiotemporal dynamics of our cortical circuit model. Model parameters are the same as in Gu et al.^15^, except for ${\tau }_{d}^{I}=5$ ms. ${N}^{E}=63\times 63=3969$ excitatory neurons and ${N}^{I}=1000$ inhibitory neurons are modeled. Each neuron is described by a 1-dimensional differential equation (i.e., the leaky integrate-and-fire model). The circuit model is composed of a large number of leaky integrate-and-fire neurons (i.e., 4969); it is thus a high-dimensional dynamical system.

Neighboring excitatory neurons (one grid unit) are separated by 7.4 μm. Simulations used the forward Euler method with a time-step of 0.1 ms. Initial membrane potentials were uniformly distributed between ${V}_{{rt}}$ = −60 mV and ${V}_{{th}}$ = −50 mV. A typical trial covered 100 s, with first 500 ms excluded. Variance (SEM) was calculated from 100 trials unless otherwise stated. All these parameters were calibrated based on the biophysics properties of neurons. Near-criticality dynamics in the transition region between the asynchronous state and the propagating wave state was characterized by the branching parameter of spikes and the susceptibility^15^, two typical quantities indexing critical transitions. In the present study, we only changed one parameter, the I-E ratio, to study the spatiotemporal nature (i.e., Lévy walk characteristic) of the emergent activity patterns.

To calculate local field potential signals in our model, we adapted the LFP proxy as used by Mazzoni et al.^79^, in which LFP was approximated as the sum of the absolute values of excitatory and inhibitory currents. This proxy was able to accurately reproduce the observed LFP power spectra. Because LFP signals can be well captured by a weighted spatial sum within a two-dimensional Gaussian window centered on the recording electrode^80^, we applied a spatial Gaussian window to the sum of absolute values of currents:

12 $$L\left({{\boldsymbol{r}}}_{e},t\right)=\mathop{\sum }\limits_{i}^{{N}^{E}}\left(\left|{I}_{i}^{{EE}}\right|+\left|{I}_{i}^{{EI}}\right|\right){e}^{-\frac{{\left({{\boldsymbol{r}}}_{i}-{{\boldsymbol{r}}}_{e}\right)}^{2}}{2{\sigma }_{L}^{2}}}$$

where ${{\boldsymbol{r}}}_{e}$ is the coordinate vector of the simulated LFP recording electrode, ${{\boldsymbol{r}}}_{i}$ is the coordinate vector of neuron $i$ and $\left|{I}_{i}^{\alpha \beta }\right|$ is the total current received by neuron $i$ in population α from population $\beta$. A spatial scale ${\sigma }_{L}$ = 8 (56–80 $\mu$m) was used, consistent with the upper estimated limit ~100 µm ^80^. Our results are not sensitive to the spatial scale. The simulated LFP signal was bandpass filtered between 1 and 1000 Hz with a 4th order Butterworth filter to model the experimentally measured broadband LFP. Synaptic currents were estimated as:

13 $${EPSC}=\left({I}_{{AMPA}}+{I}_{{ext}}\right)\frac{{V}_{I}-{V}_{E}}{V\left(t\right)-{V}_{E}}$$

14 $${IPSC}={I}_{{GABA}}\frac{{V}_{E}-{V}_{I}}{V\left(t\right)-{V}_{I}}$$

where ${I}_{{AMPA}}$ and ${I}_{{GABA}}$ are interior excitatory and inhibitory synaptic currents, ${I}_{{ext}}$ is external excitatory current, ${V}_{I}$ and ${V}_{E}$ are the inhibitory and excitatory synaptic reversal potentials, and $V\left(t\right)$ is the time-varying membrane potential.

### Statistics and reproducibility

The results were expressed as mean $\pm$ standard deviation (s.d.). The experimental data were analyzed and compared against surrogate data using Wilcoxon rank sum test. Fifty surrogate datasets were generated and compared. The models were selected based on a Vuong test. The *p* values < 0.05 were considered to be significance.

### Reporting summary

Further information on research design is available in the Nature Research Reporting Summary linked to this article.

## Supplementary information

Supplementary Information

Reporting Summary

## Data Availability

The datasets generated during and/or analyzed during the current study are available from the corresponding author on reasonable request.

## Code availability

All analysis scripts that reproduce the results of the present study are readily accessible to and open for reuse by the reader: https://github.com/BrainDynamicsUSYD/NeuroFdpToolbox (10.5281/zenodo.4732266)
