# Supplementary material for: Lévy walk dynamics explain gamma burst patterns in primate cerebral cortex
Source: Commun Biol. 2021 Jun 15;4:739. doi: 10.1038/s42003-021-02256-1 (PMC8206356; doi:10.1038/s42003-021-02256-1)
Supplement: Supplementary file 1 — Supplementary Information [file 42003_2021_2256_MOESM1_ESM.pdf]

Supplementary Information

Lévy walk dynamics explain gamma burst patterns in primate cerebral cortex

Yuxi Liu[1,2¶], Xian Long[1,2¶], Paul R. Martin[2,3,4], Samuel G. Solomon[5] and Pulin Gong[1,2*]

[1] School of Physics, University of Sydney, New South Wales, 2006, Australia

[2] ARC Centre of Excellence for Integrative Brain Function, University of Sydney, 2001, New South Wales, Australia

[3] Discipline of Physiology, University of Sydney, Sydney, New South Wales, 2006, Australia

[4] Save Sight Institute, University of Sydney, Sydney, New South Wales, 2001, Australia

[5] Department of Experimental Psychology, University College London, London, WC1P 0AH, United Kingdom

[*] pulin.gong@sydney.edu.au

[¶] Equal contribution.

Supplementary Note

When a localized gamma pattern moves around a given location, bursts are often detected on more than one electrode at consecutive time steps. The centre of mass of the burst pattern can therefore move a distance which is less than the distance between electrodes. To demonstrate that these small movements are not noise, but rather are part of the Lévy walk process, we tested different minimal threshold values of step lengths from 80 microns to 400 microns and found that for these cases, the CCPDs of step lengths (1D model) show similar truncated power law distributions (Fig. S5A, S5B). Additionally, we found that the CCPD of step length of gamma patterns without spatial filtering shows a heavy tail distribution and follows the truncated power law distribution (Fig. S5C, S5D).

Supplementary Figures

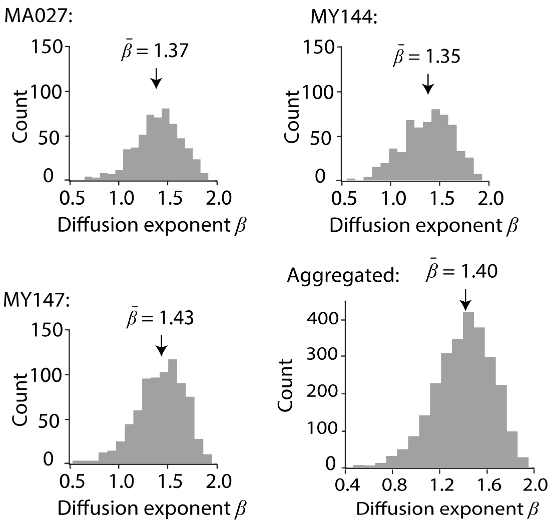

Figure S1. Distribution of diffusion exponent β of gamma burst patterns of each animal (MA027, MY144 and MY147) and collapsed for 4 animals (20 minutes in total); the mean values of β are 1.37, 1.35, 1.43, and 1.40, respectively.

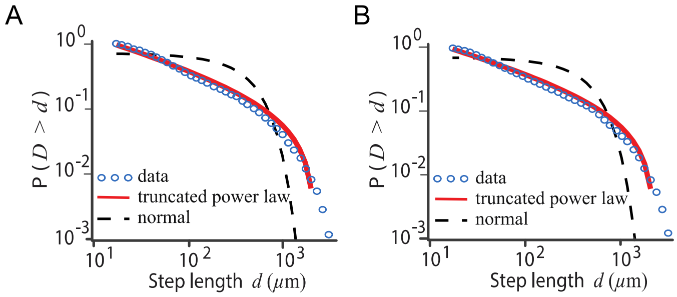

Figure S2. CCPD of step length (left: 1D model; right: 2D model with $\theta = 40^{o}$) of the aggregated data (of all animals). Red line indicates a fitted truncated power distribution with λ =1.23 and λ =1.48, respectively. For comparison, a normal distribution fit (black line) is shown.

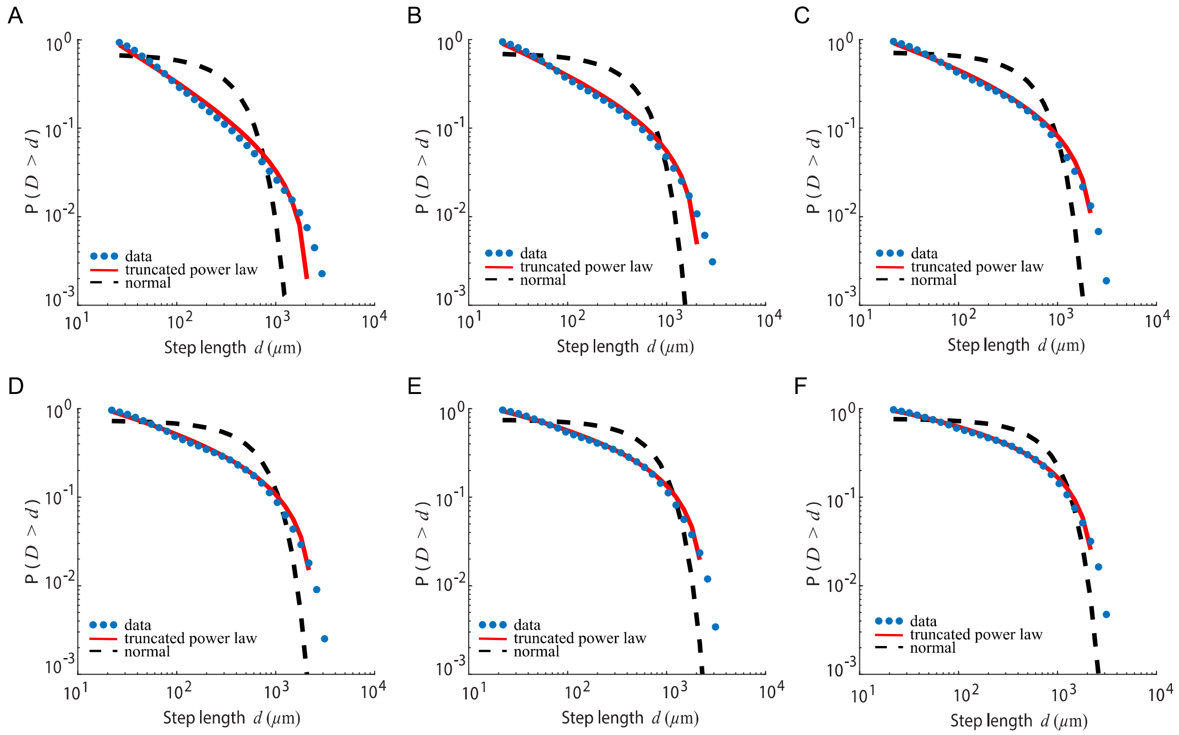

Figure S3. CCPD of step length defined by different turning angles ($\theta = 20^o$ to 120° with increment of 20°; $\theta = 20^o, 40^o, 60^o$ for the first row and $\theta = 80^o, 100^o, 120^o$ for the second row) of MA026. Red lines indicate fitted truncated power distributions with λ =1.67, λ =1.45, λ =1.35, λ =1.24, λ =1.15 and λ =1.05, respectively. For comparison, a normal distribution fit (black line) is shown.

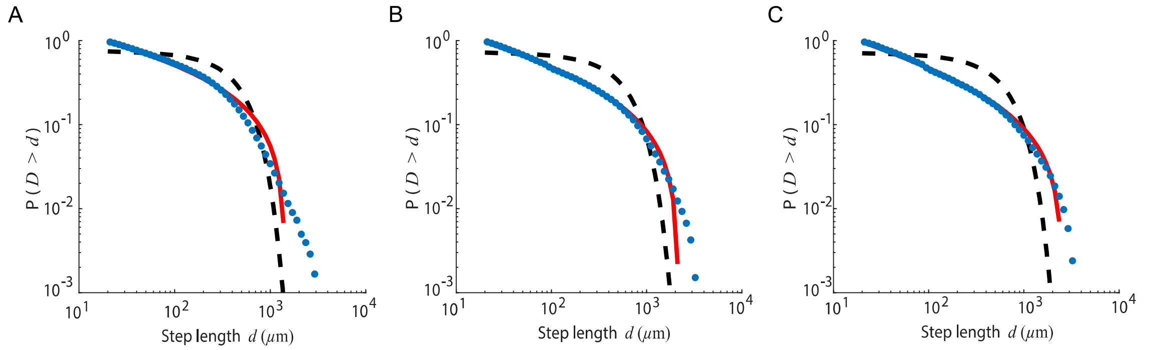

Figure S4. CCPD (1D model) of gamma burst patterns defined by different threshold (A: s.d. = 1.5; B: s.d. = 2; C: s.d. = 2.5) of animal MA026. Red line indicates a fitted truncated power distribution with λ =1.1, λ =1.22 and λ =1.28 respectively. For comparison, a normal distribution fit (black line) is shown.

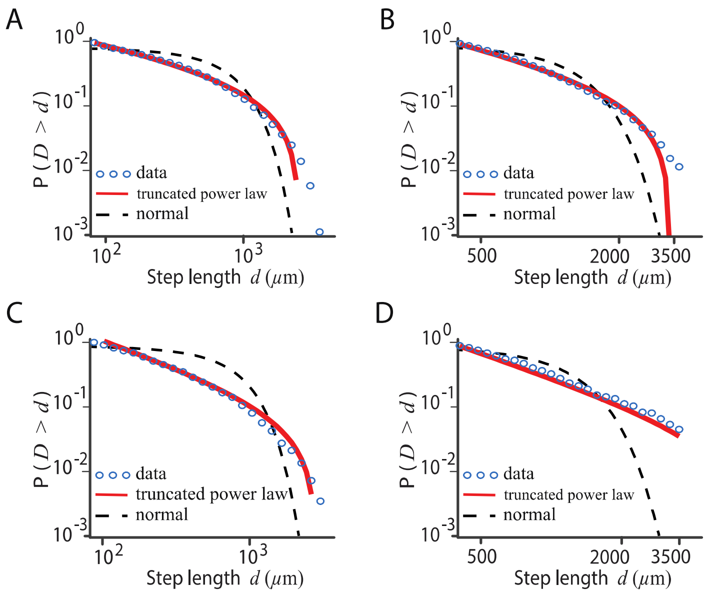

Figure S5. CCPD of step length (1D model) of MA026 with minimal displacement threshold 80 μm (A) and 400 μm (B). CCPD of step length (1D model) of MA026 with no spatial filtering and with minimal displacement threshold 80 μm (C) and 400 μm (D). Red line indicates a fitted truncated power distribution with λ =1.38, λ =1.68, λ =1.5 and λ =2.25, respectively. For comparison, a normal distribution fit (black line) is shown.

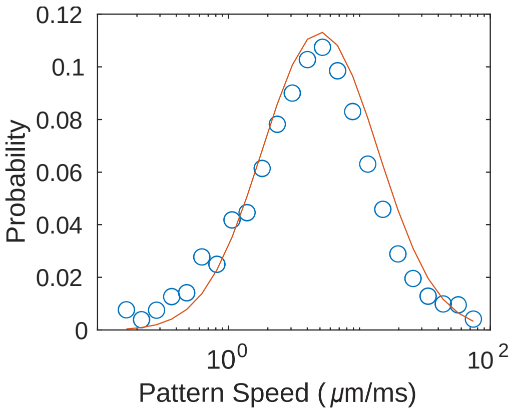

Figure S6. Distribution of the propagation speed of localized activity patterns in State II (blue circles). Red line indicates a fitted log-normal distribution.

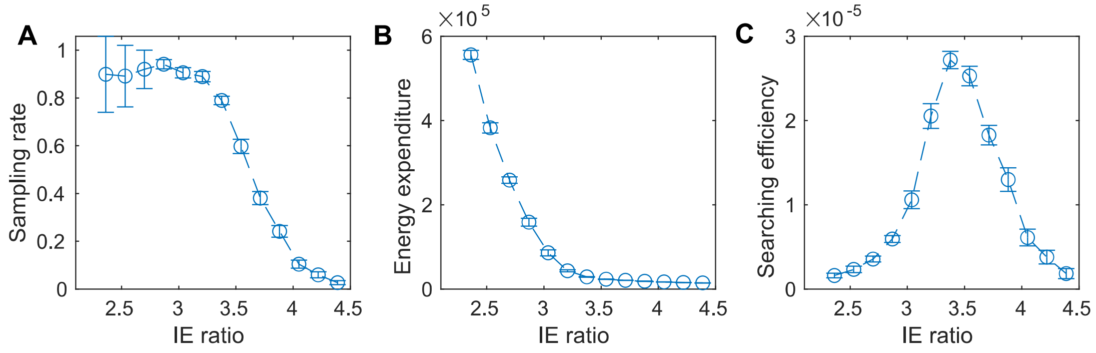

Figure S7. Left: Sampling rate of the localized activity pattern as a function of the I-E ratio. Middle: Change of the energy expenditure with different I-E ratios. Right: Searching efficiency of the activity pattern as a function of the I-E ratio.

Table S1. Akaike weights of different model fittings and MLE of truncated power law exponent λ for each animal

Animal	Model	Truncated power law	Exponential	Normal	Log-normal	Gamma	λ
MA026	$\theta = 40^o$	1.00	0.00	0.00	0.00	0.00	1.45
	$\theta = 80^o$	1.00	0.00	0.00	0.00	0.00	1.24
	$\theta = 120^o$	1.00	0.00	0.00	0.00	0.00	1.05
	1D model	1.00	0.00	0.00	0.00	0.00	1.32
MA027	$\theta = 40^o$	1.00	0.00	0.00	0.00	0.00	1.52
	$\theta = 80^o$	1.00	0.00	0.00	0.00	0.00	1.27
	$\theta = 120^o$	1.00	0.00	0.00	0.00	0.00	1.09
	1D model	1.00	0.00	0.00	0.00	0.00	1.33
MY144	$\theta = 40^o$	1.00	0.00	0.00	0.00	0.00	1.48
	$\theta = 80^o$	1.00	0.00	0.00	0.00	0.00	1.25
	$\theta = 120^o$	1.00	0.00	0.00	0.00	0.00	1.08
	1D model	1.00	0.00	0.00	0.00	0.00	1.33
MY147	$\theta = 40^o$	1.00	0.00	0.00	0.00	0.00	1.44
	$\theta = 80^o$	1.00	0.00	0.00	0.00	0.00	1.21
	$\theta = 120^o$	1.00	0.00	0.00	0.00	0.00	1.02
	1D model	1.00	0.00	0.00	0.00	0.00	1.30

Table S2. Akaike weights of different model fittings and MLE of truncated power law exponent λ in simulation data

Model	Truncated power law	Exponential	Normal	Log-normal	Gamma	λ
$\theta = 20^o$	1.00	0.00	0.00	0.00	0.00	1.26
$\theta = 40^o$	1.00	0.00	0.00	0.00	0.00	1.13
$\theta = 60^o$	1.00	0.00	0.00	0.00	0.00	1.01
1D model	1.00	0.00	0.00	0.00	0.00	1.32
