# Supplementary material for: Lévy walk dynamics explain gamma burst patterns in primate cerebral cortex
Source: Commun Biol. 2021 Jun 15;4:739. doi: 10.1038/s42003-021-02256-1 (PMC8206356; doi:10.1038/s42003-021-02256-1)
Supplement: Supplementary file 2 — Reporting Summary [file 42003_2021_2256_MOESM2_ESM.pdf]

nature research

Corresponding author(s): Pulin Gong

Last updated by author(s): 18/05/21

Reporting Summary

Nature Research wishes to improve the reproducibility of the work that we publish. This form provides structure for consistency and transparency in reporting. For further information on Nature Research policies, see our Editorial Policies and the Editorial Policy Checklist.

Statistics

For all statistical analyses, confirm that the following items are present in the figure legend, table legend, main text, or Methods section.

n/a	Confirmed	
☐	☒	The exact sample size (n) for each experimental group/condition, given as a discrete number and unit of measurement
☒	☐	A statement on whether measurements were taken from distinct samples or whether the same sample was measured repeatedly
☐	☒	The statistical test(s) used AND whether they are one- or two-sided<br>*Only common tests should be described solely by name; describe more complex techniques in the Methods section.*
☒	☐	A description of all covariates tested
☒	☐	A description of any assumptions or corrections, such as tests of normality and adjustment for multiple comparisons
☐	☒	A full description of the statistical parameters including central tendency (e.g. means) or other basic estimates (e.g. regression coefficient) AND variation (e.g. standard deviation) or associated estimates of uncertainty (e.g. confidence intervals)
☐	☒	For null hypothesis testing, the test statistic (e.g. F, t, r) with confidence intervals, effect sizes, degrees of freedom and P value noted<br>*Give P values as exact values whenever suitable.*
☒	☐	For Bayesian analysis, information on the choice of priors and Markov chain Monte Carlo settings
☒	☐	For hierarchical and complex designs, identification of the appropriate level for tests and full reporting of outcomes
☐	☒	Estimates of effect sizes (e.g. Cohen's d, Pearson's r), indicating how they were calculated

Our web collection on statistics for biologists contains articles on many of the points above.

Software and code

Policy information about availability of computer code

Data collection	Electrophysiology data were amplified and digitized by an RZ2 real-time processor (Tucker-Davis Technologies).
Data analysis	Model simulation and electrophysiology data analysis was written in MATLAB (version 2020a) and C++.

For manuscripts utilizing custom algorithms or software that are central to the research but not yet described in published literature, software must be made available to editors and reviewers. We strongly encourage code deposition in a community repository (e.g. GitHub). See the Nature Research guidelines for submitting code & software for further information.

Data

Policy information about availability of data

All manuscripts must include a data availability statement. This statement should provide the following information, where applicable:

- Accession codes, unique identifiers, or web links for publicly available datasets
- A list of figures that have associated raw data
- A description of any restrictions on data availability

MATLAB and C++ codes were used to simulate the model and analyze the data. They can be found at the following GitHub repository (repo): https://github.com/BrainDynamicsUSYD/NeuroFdpToolbox. All data are available from the corresponding authors upon request.

Field-specific reporting

Life sciences study design

All studies must disclose on these points even when the disclosure is negative.

Sample size	Four marmoset monkeys were used for this study. No sample size calculation was performed. The number used conforms to standards in the field for non-human primate electro-physiology experiments.
Data exclusions	To analyze data with equal length, the first 300 second of data of spontaneous activity were kept. In the signal processing, the first and the last two seconds of data were discarded to reduce the boundary effect generated by the filtering process.
Replication	All of the reported findings were replicated using the same methodology in all the four monkeys.
Randomization	Randomization was not relevant for this study as there are no experimental groups.
Blinding	Blinding was not relevant to this study as no experimental group was defined.

Reporting for specific materials, systems and methods

We require information from authors about some types of materials, experimental systems and methods used in many studies. Here, indicate whether each material, system or method listed is relevant to your study. If you are not sure if a list item applies to your research, read the appropriate section before selecting a response.

Materials & experimental systems

n/a	Involved in the study	
☒	☐	Antibodies
☒	☐	Eukaryotic cell lines
☒	☐	Palaeontology and archaeology
☐	☒	Animals and other organisms
☒	☐	Human research participants
☒	☐	Clinical data
☒	☐	Dual use research of concern

Methods

n/a	Involved in the study	
☒	☐	ChIP-seq
☒	☐	Flow cytometry
☒	☐	MRI-based neuroimaging

Animals and other organisms

Policy information about studies involving animals; ARRIVE guidelines recommended for reporting animal research

Laboratory animals	Four adult male marmosets (Callithrix jacchus) were used in this study.
Wild animals	The study did not involve wild animals.
Field-collected samples	The study did not involve samples collected from the field.
Ethics oversight	Marmosets were obtained from the Australian National Health and Medical Research Council (NHMRC) combined breeding facility. Procedures were approved by Institutional (University of Sydney) Animal Ethics Committee and conform to the Society for Neuroscience and NHMRC policies on the use of animals in neuroscience research.

Note that full information on the approval of the study protocol must also be provided in the manuscript.
